# The contribution of emotional empathy to approachability judgments assigned to emotional faces is context specific

**DOI:** 10.3389/fpsyg.2015.01209

**Published:** 2015-08-19

**Authors:** Megan L. Willis, Danielle L. Lawson, Nicole J. Ridley, Peter Koval, Peter G. Rendell

**Affiliations:** ^1^School of Psychology, Australian Catholic University, Strathfield, NSW, Australia; ^2^Australian Research Council Centre of Excellence in Cognition and its Disorders, Australian Catholic University, Sydney, NSW, Australia; ^3^School of Psychology, Australian Catholic University, Melbourne, VIC, Australia; ^4^Faculty of Psychology and Educational Sciences, University of Leuven, Leuven, Belgium

**Keywords:** emotional empathy, approach/avoidance, facial expression, prosocial behavior, situational context, social behavior, emotion

## Abstract

Previous research on approachability judgments has indicated that facial expressions modulate how these judgments are made, but the relationship between emotional empathy and context in this decision-making process has not yet been examined. This study examined the contribution of emotional empathy to approachability judgments assigned to emotional faces in different contexts. One-hundred and twenty female participants completed the questionnaire measure of emotional empathy. Participants provided approachability judgments to faces displaying angry, disgusted, fearful, happy, neutral, and sad expressions, in three different contexts—when evaluating whether they would approach another individual to: (1) receive help; (2) give help; or (3) when no contextual information was provided. In addition, participants were also required to provide ratings of perceived threat, emotional intensity and label facial expressions. Emotional empathy significantly predicted approachability ratings for specific emotions in each context, over and above the contribution of perceived threat and intensity, which were associated with emotional empathy. Higher emotional empathy predicted less willingness to approach people with angry and disgusted faces to receive help, and a greater willingness to approach people with happy faces to receive help. Higher emotional empathy also predicted a greater willingness to approach people with sad faces to offer help, and more willingness to approach people with happy faces when no contextual information was provided. These results highlight the important contribution of individual differences in emotional empathy in predicting how approachability judgments are assigned to facial expressions in context.

## Introduction

In everyday social settings, we are constantly required to make split second decisions about whether to approach or avoid other individuals, from deciding who to approach for directions when we are lost, to whether to stop and offer assistance to an individual in need. When an individual is unknown to us, we are capable of making these kind of first impressions from another’s facial appearance rapidly, with different individuals showing a high degree of consistency in the precise judgments they assign to specific faces (Adolphs et al., 1998; Willis and Todorov, 2006). Recent research has demonstrated that the social judgment of approachability appears to be driven largely by an individual’s facial expression (Willis et al., 2011a,b). Faces depicting negative emotions (i.e., anger, disgust, fear, and sadness) are consistently rated as less approachable than faces depicting happy or neutral expressions. Distinct differences are also seen within valence categories, with angry and disgusted faces consistently rated as less approachable than sad and fearful faces (Porter et al., 2007; Willis et al., 2011b). These differences in perceived approachability that emerge between negatively valenced emotions are assumed to reflect differences in the direct threat conveyed by an expression (Adolphs et al., 1998; Willis et al., 2011a). This judgment is particularly important for successful social navigation, given that deficits in the ability to accurately judge the appropriateness of engaging with another individual could have dramatic ramifications for the individual’s wellbeing (Blair, 2003). Indeed, impairments in these social judgments have been observed in individuals within several clinical populations, including people with bilateral amygdala lesions, autism spectrum disorder, schizophrenia, and Williams syndrome, who have been noted to demonstrate socially inappropriate, and in extreme cases, risky behavior (Adolphs et al., 1998; Bellugi et al., 1999; Hall et al., 2004; Frigerio et al., 2006).

While an individual’s emotion appears to be a key factor driving the perception of approachability, it is clear that individuals differ markedly in their willingness to approach individuals when faced with the same situation, whether it is approaching another person to ask for assistance, or to offer help to a person in need. What is less well understood is what underlying traits may account for these individual differences in social behavior. One trait that may play an important role in influencing the way in which we respond to emotion in others is that of empathy. Researchers define empathy as a psychological construct, which involves both cognitive and affective components; *emotional empathy* referring to emotional contagion (e.g., “I feel what you feel”) and *cognitive empathy* involving the process of understanding another person’s perspective (e.g., “I understand what you feel”; Shamay-Tsoory et al., 2009). Self-reported emotional empathy is associated with a heightened capacity to recognize facial expressions (Mayer et al., 1990; Carr et al., 2003; Blair, 2005). This advantage appears to be most evident in difficult facial expression tasks (e.g., when faces are presented only briefly; Besel and Yuille, 2010) and for the facial expression of fear (Carr et al., 2003; Besel and Yuille, 2010). In addition, individuals with higher levels of emotional empathy perceive facial expressions as more emotionally intense and this increased perception of emotional intensity is understood to be associated with heightened physiological responses to emotional faces (Blair, 2005; Gery et al., 2009; Dimberg et al., 2011).

The perception of emotional intensity is understood to be one factor that influences the type of approachability judgments assigned to emotional faces. For instance, angry and disgusted faces, which are deemed the most unapproachable, are perceived as more emotionally intense (i.e., angry and disgusted faces) than negative emotions of lesser intensity (i.e., sad and fearful faces). Whereas happy faces, which are considered the most approachable, are rated as more emotionally intense than fearful, neutral, and sad faces (Willis et al., 2011b). Given that individuals with higher levels of emotional empathy are thought to perceive emotion more intensely, one might expect that emotional empathy would modulate the perception of approachability from emotional faces, such that individuals with higher levels of emotional empathy are likely to be more sensitive to facial expressions when judging the approachability of emotional faces. Thus, the current study sought to investigate the relationship between emotional empathy and approachability judgments assigned to emotional faces.

In addition to influencing the perception of emotion, emotional empathy is also understood to influence an individual’s willingness to engage in *prosocial behavior*, which is defined by Eisenberg and Miller (1987) as voluntary and intentional behavior that yields benefits for others. Empathy has long been suggested to be the most likely motivator of prosocial behavior and altruism (Eisenberg and Miller, 1987; Batson, 1998). More specifically, individuals higher in self-reported emotional empathy are more likely to respond prosocially than those with lower empathy (Mehrabian and Epstein, 1972; Balconi and Bortolotti, 2012; Paciello et al., 2013). Moreover, prosocial behavior is associated with superior fear recognition (Marsh et al., 2007), which may indicate that the capacity to accurately detect distress cues (e.g., emotions of fear and sadness) may increase an individual’s likelihood of behaving prosocially. As enhanced emotional empathy levels have been linked to an advantage for recognition of fearful facial expressions (Besel and Yuille, 2010), it follows that individuals higher in emotional empathy may have an enhanced capacity to recognize distress cues and therefore be more inclined to engage in prosocial behaviors when exposed to such distress.

It is thought that individual differences in prosocial behavior are the most apparent in negative situations, where prosocial behaviors may be warranted (Balconi and Canavesio, 2013). Specifically, highly empathetic people are more likely to help if the problem and their role in the solution is clearly identified (Einolf, 2008), and if it is perceived that help is needed (Penner et al., 2005). The way that empathy influences prosocial responding may also vary depending on the type of emotional cue (e.g., type of facial expression). This is consistent with the behavioral ecology view promoted by Fridlund (1994) that describes expressions as tools that help perceivers understand the intentions and likely actions of the expresser. For instance, fearful faces are thought to signal the presence of a significant, yet undetermined source of danger in the environment and communicate that the individual is in distress, while sadness reflects a desire to appease and may be used to elicit sympathy from the social group (Hasson, 1997). The influence of facial expressions on behavioral responses was demonstrated by Small and Verrochi (2009), who indicated that emotions that are consistent with needing help are more likely to elicit help from empathetic people. Specifically, they found that charity advertisements depicting sad faces were more likely to generate the most donations, compared to neutral or happy faces. Telle and Pfister (2012) found that congruence of person and situation-related information, in particular perceived negative affect (e.g., sadness) in response to a negative event, was most likely to elicit prosocial behavior, promoting the relevance that context has in interpretation of facial expressions.

The results outlined above suggest that individuals higher in emotional empathy may be more likely to approach others to help when the need for help is clear, and the emotion communicated is appropriate, relative to those with lower levels of empathy. However, whether empathy promotes approach-related behaviors in just this specific context, or whether empathy modulates approachability judgments in all contexts has not yet been examined. For instance, it has been proposed that individuals are more likely to approach others if they have power (e.g., have the option to provide help or information to others) and are more vigilant to markers of threat if they lack power (e.g., need assistance; Keltner et al., 2003). If empathy is indeed related to enhanced recognition and increased perceived intensity of facial expressions, it is possible that in a context where the individual *needs* help, that those higher in empathy are more sensitive to facial expressions that signal threat or danger—and would be less inclined to approach individuals showing these expressions. However, this has not yet been empirically tested. Therefore, in addition to examining the relationship between emotional empathy and the perception of approachability from emotional faces, we were also interested in how the contribution of emotional empathy interacts with the particular situational context in which discrete emotional faces are encountered.

The aim of the current study was to examine the relationship between emotional empathy and context on judgments of approachability to emotional faces. In the current study, all participants completed a self-report measure of emotional empathy. We contrasted approachability judgments assigned to emotional faces (i.e., angry, disgusted, fearful, happy, sad, as well as a neutral pose) across three approachability tasks: (1) when no contextual information was provided; (2) when the observer was asked to evaluate whether they would approach to receive help; and (3) when asked whether they would approach to provide help. Based on the previous research reviewed, we hypothesized that higher empathy would predict a greater willingness to approach faces depicting distress-related emotions (i.e., sadness and fear) in the giving help context. Given the anticipated relationship between empathy and perceived intensity of emotional faces, we also hypothesized that higher empathy would predict more extreme judgments across contexts for the emotions previously rated as most intense (i.e., higher approachability ratings for happy expressions, and lower approachability ratings for disgusted and angry expressions). In order to develop a comprehensive understanding of the relationship between emotional empathy and judgments of approachability to emotional faces, participants were also required to provide ratings of perceived threat, emotional intensity and label the facial expressions.

## Materials and Methods

### Ethics Statement

This research was approved by Australian Catholic University’s Human Research Ethics Committee (HREC). All participants provided written informed consent to participate in the study.

### Participants

One-hundred and twenty females were recruited from the general population and from the undergraduate psychology student population of the Australian Catholic University. Participants received either course credit or entry into a prize draw for their participation. Ages ranged from 18 to 67 years (*M* = 27.62, SD = 12.68). All participants had normal or corrected-to-normal vision and no history of brain injury. Only female participants were recruited for the study to control for the documented sex differences on our measure of emotional empathy (i.e., females score significantly higher than males), the *questionnaire measure of emotional empathy* (QMEE; Mehrabian and Epstein, 1972), as well as differences in facial expression processing that have been observed between the sexes (see Dimberg, 1997; Hermans et al., 2006; Luo et al., 2014).

### Stimuli

Faces of 10 individuals (five female) were sourced from the Karolinska Directed Emotional Faces (KDEF) database (Lundqvist et al., 1998). Photographs of each individual displaying an angry, disgusted, fearful, happy, sad, and neutral pose were selected for a total of 60 faces. The faces (256 gray levels, 72 ppi) were scaled to be the same size and were displayed within a black rectangular background of 6.5 cm × 8.8 cm, which subtended a visual angle of approximately 6.20° by 8.39° at the experimental resolution.

### Approachability Tasks

#### Neutral Context

In this task participants judged the approachability of the 60 faces described above. They were not given any contextual information upon which to base their judgment. For each face, they were instructed to indicate their agreement with the statement “I would approach this person.” The faces were shown one at a time on a white background, in a randomized order. Participants indicated their agreement with the statement on a 9-point Likert scale ranging from –4 (Strongly disagree) to +4 (Strongly agree). The stimulus was presented in the middle of the screen with the statement presented above the face and the response scale presented below the face. The stimulus, scale and statement remained on the screen until a response was made via mouse click. An inter-trial interval of 500 ms preceded the onset of the next trial. The neutral context was included to provide a baseline measurement of approachability ratings in order to more completely understand the importance of context in driving approachability judgments.

#### Receiving Help Context

The receiving help context has been used in our previous research (Willis et al., 2010, 2011a,b, 2013). Participants were asked to imagine being in a situation where they are on a crowded street on their way to meet a friend. They were told to imagine that they were lost and in a hurry, and needed to ask someone for directions in order to meet their friend on time. They were asked to imagine seeing each person in a crowd and to indicate the extent to which they agreed with following statement “I would approach this person to look for directions.” The procedure was otherwise identical to the neutral context task.

#### Giving Help Context

The giving help context has been used previously (Willis et al., 2015a,b). In this task, participants were asked to imagine leaving their local library and seeing a person carrying a pile of books trip and drop the books. For each face, participants were asked to indicate their agreement with the statement “I would approach this person and offer them help.” The task was otherwise identical to the two other approachability tasks.

### Threat Perception Task

In this task, participants were asked to rate how threatening they found each face. Responses were made on a 9-point Likert scale from 0 (Not at all threatening) to 8 (Extremely threatening). The response scale was presented underneath each image. The presentation of stimuli, method of response, and inter-trial interval were as described for the approachability tasks.

### Emotion Recognition and Intensity Rating Task

In this task, participants were shown the faces again and were asked to categorize each facial expression from the six options displayed below each face; angry, disgusted, fearful, happy, neutral, and sad. After labeling the expression, participants were then asked to indicate how intensely the emotion was portrayed on a 9-point Likert scale, ranging from 0 (Not at all intense) to 8 (Extremely intense). As with the other tasks, an inter-trial interval of 500 ms preceded the onset of the next face.

### Empathy Scale

Emotional empathy was assessed using the QMEE developed by Mehrabian and Epstein (1972). This is a self-report questionnaire of 33 statements designed to assess typical empathic emotional response (e.g., “I am able to make decisions without being influenced by people’s feelings”). Participants rated the degree to which they agreed that each statement applied to them on a 9-point Likert scale from –4 (Strongly disagree) to +4 (Strongly agree). The QMEE scale had very good internal reliability, Cronbach’s α = 0.82, in the current sample. Empathy scores ranged from –67.00 to 124.00 (*M* = 44.48, SD = 27.39).

### Procedure

At the commencement of the study, participants provided demographic information and completed the QMEE. The approachability tasks were then completed, with the neutral context task completed first by all participants in order to ensure that responses were not confounded by the other contexts. The giving help context and receiving help context tasks were then completed in a counterbalanced order between participants. Participants then completed the threat perception task, followed by the emotion recognition and intensity rating task. Stimulus presentation was controlled using Superlab (Cedrus Corporation) and viewed on a 27-inch iMac computer.

### Statistical Analyses

Mean approachability ratings were first analyzed using a 3 × 6 repeated measures ANCOVA with *context* (neutral context, giving help, receiving help) and *emotion* (angry, disgusted, fearful, happy, neutral, sad) as within-subjects factors and standardized empathy scores as a continuous covariate. The Greenhouse–Geisser epsilon adjusted value is reported in all instances where the sphericity assumption was violated. Multiple regressions were then performed in order to investigate significant interactions with emotion and empathy scores, separately for each context. We also performed correlations between empathy scores and threat ratings, intensity ratings and labeling accuracy to guide their inclusion as predictors in the multiple regressions that were performed.

## Results

Table 1 displays descriptive statistics for threat ratings, intensity ratings and labeling accuracy, along with correlations between empathy and each measure, separately for each emotion. As Table 1 shows, higher levels of emotional empathy were significantly associated with the perception of angry, disgusted and fearful faces as more threatening. Higher emotional empathy was also significantly associated with perception of disgusted, fearful, happy and sad faces and more emotionally intense. Emotional empathy was not associated with heightened labeling accuracy for any emotion.

**TABLE 1:** **Descriptive statistics for threat, intensity and accuracy for each emotion and zero-order correlations with empathy scores**.

****	**Threat**		**Intensity**		**Accuracy**	
	***M* (SE)**	***r* (198)**	***M* (SE)**	***r* (198)**	***M* (SE)**	***r* (198)**
Angry	5.04 (0.15)	0.21*	5.42 (0.10)	0.17	9.36 (0.09)	0.13
Disgusted	4.04 (0.16)	0.24*	5.86 (0.10)	0.26**	7.77 (0.19)	0.08
Fearful	2.54 (0.13)	0.22*	5.42 (0.10)	0.24*	8.78 (0.16)	0.05
Sad	2.05 (0.14)	0.13	4.99 (0.11)	0.25*	9.02 (0.12)	0.13
Neutral	2.41 (0.13)	0.16	4.19 (0.15)	0.02	9.04 (0.13)	0.00
Happy	0.56 (0.07)	–0.01	5.49 (0.15)	0.20*	9.80 (0.06)	0.00

*p < 0.05. **p < 0.005.

### Approachability

An initial 3 (context) × 6 (emotion) repeated measures ANCOVA with standardized empathy scores as a continuous covariate revealed significant main effects of context, *F*(1.59,188.03) = 69.34, *p* < 0.001, ${\text{η}}_{p}^{2}$ = 0.37, and emotion, *F*(2.93,346.29) = 731.54, *p* < 0.001, ${\text{η}}_{p}^{2}$ = 0.86, as well as significant two-way interactions for Emotion × Empathy, *F*(2.93,346.29) = 4.55, *p* = 0.004, ${\text{η}}_{p}^{2}$ = 0.04, Context × Emotion, *F*(4.61,544.19) = 55.61, *p* < 0.001, ${\text{η}}_{p}^{2}$ = 0.32, and a marginally significant Context × Empathy interaction, *F*(1.59,188.03) = 3.03, *p* = 0.062, ${\text{η}}_{p}^{2}$ = 0.03. The three-way Context × Emotion × Empathy interaction was also significant, *F*(4.61,544.19) = 3.83, *p* = 0.003, ${\text{η}}_{p}^{2}$ = 0.03. The main effect of empathy was not significant, *F*(1,118) = 0.06, *p* = 0.811, ${\text{η}}_{p}^{2}$ = 0.00.

We decomposed the significant three-way Context × Emotion × Empathy Group interaction by running three separate repeated measures ANCOVAs, one for each context, with emotion as the within-subjects factor and standardized empathy scores as a continuous covariate. For the neutral context, there was a significant main effect of emotion, *F*(3.37,397.64) = 383.68, *p* < 0.001, ${\text{η}}_{p}^{2}$ = 0.76, but no significant main effect of empathy, *F*(1,118) = 0.25, *p* = 0.615, ${\text{η}}_{p}^{2}$ = 0.00. However, a significant Emotion × Empathy interaction emerged, *F*(3.37,397.64) = 2.74, *p* = 0.037, ${\text{η}}_{p}^{2}$ = 0.02. In the receiving help context, the main effect of emotion was again significant, *F*(3.06,360.76) = 930.67, *p* < 0.001, ${\text{η}}_{p}^{2}$ = 0.89, and the main effect of empathy was marginally significant, *F*(1,118) = 3.89, *p* = 0.051, ${\text{η}}_{p}^{2}$ = 0.03. These main effects were qualified by a significant Emotion × Empathy interaction, *F*(3.06,360.76) = 7.31, *p* < 0.001, ${\text{η}}_{p}^{2}$ = 0. 06. Finally, in the giving help context, the main effect of emotion was significant, *F*(2.71,320.36) = 252.98, *p* < 0.001, ${\text{η}}_{p}^{2}$ = 0.68, as was the Emotion × Empathy interaction, *F*(2.71,320.36) = 3.80, *p* = 0.013, ${\text{η}}_{p}^{2}$ = 0.03. The main effect of empathy was not significant, *F*(1,118) = 1.42, *p* = 0.235, ${\text{η}}_{p}^{2}$ = 0.01.

To probe the significant Emotion × Empathy interactions that emerged in each context, we ran a series of regressions, with empathy scores as a predictor of approachability ratings, separately for each emotion within each context. Given that empathy scores were correlated with threat and intensity judgments (see Table 1), we also included threat and intensity as simultaneous predictors of approachability, to determine whether empathy was uniquely associated with approachability ratings after controlling for the effects of these secondary variables^1^. Tables 2–4 show the inferential statistics, the unstandardized regression coefficients (*B*), the standard error (SE), and the standardized regression coefficients (β) for the regression model for each analysis.

**TABLE 2:** **Multiple regressions predicting approachability ratings in the neutral context for each emotion**.

****	**Angry**				**Disgusted**				**Fearful**				**Sad**				**Neutral**				**Happy**			
**Model**	***F***	***df***	***p***	***R*^2^**	***F***	***df***	***p***	***R*^2^**	***F***	***df***	***p***	***R*^2^**	***F***	***df***	***p***	***R*^2^**	***F***	***df***	***p***	***R*^2^**	***F***	***df***	***p***	***R*^2^**
	7.02	3,116	<0.001	0.15	6.03	3,116	0.001	0.13	0.90	3,116	0.441	0.02	3.37	3,116	0.021	0.08	7.42	3,116	<0.001	0.16	12.48	3,116	<0.001	0.24

**Predictor**	***B* (SE)**		**β**	***p***	***B* (SE)**		**β**	***p***	***B* (SE)**		**β**	***p***	***B* (SE)**		**β**	***p***	***B* (SE)**		**β**	***p***	***B* (SE)**		**β**	***p***
Intercept	–0.56 (0.63)				0.74 (0.72)				0.26 (0.74)				0.27 (0.67)				0.62 (0.39)				2.34 (0.30)			
Empathy	0.00 (0.00)		–0.03	0.723	0.01 (0.01)		0.09	0.298	0.01 (0.01)		0.09	0.341	0.01 (0.01)		0.11	0.237	0.00 (0.00)		0.06	0.500	0.01 (0.00)		0.26	0.002
Intensity	0.08 (0.13)		0.06	0.527	–0.21 (0.09)		–0.23	0.016	–0.07 (0.13)		–0.05	0.622	0.03 (0.13)		0.02	0.824	0.16 (0.07)		0.21	0.016	0.06 (0.05)		0.10	0.234
Threat	–0.35 (0.08)		–0.41	<0.001	–0.31 (0.13)		–0.22	0.023	–0.15 (0.10)		–0.14	0.148	–0.31 (0.10)		–0.27	0.003	–0.31 (0.08)		–0.33	<0.001	–0.50 (0.11)		–0.38	<0.001

**FIGURE 1:**
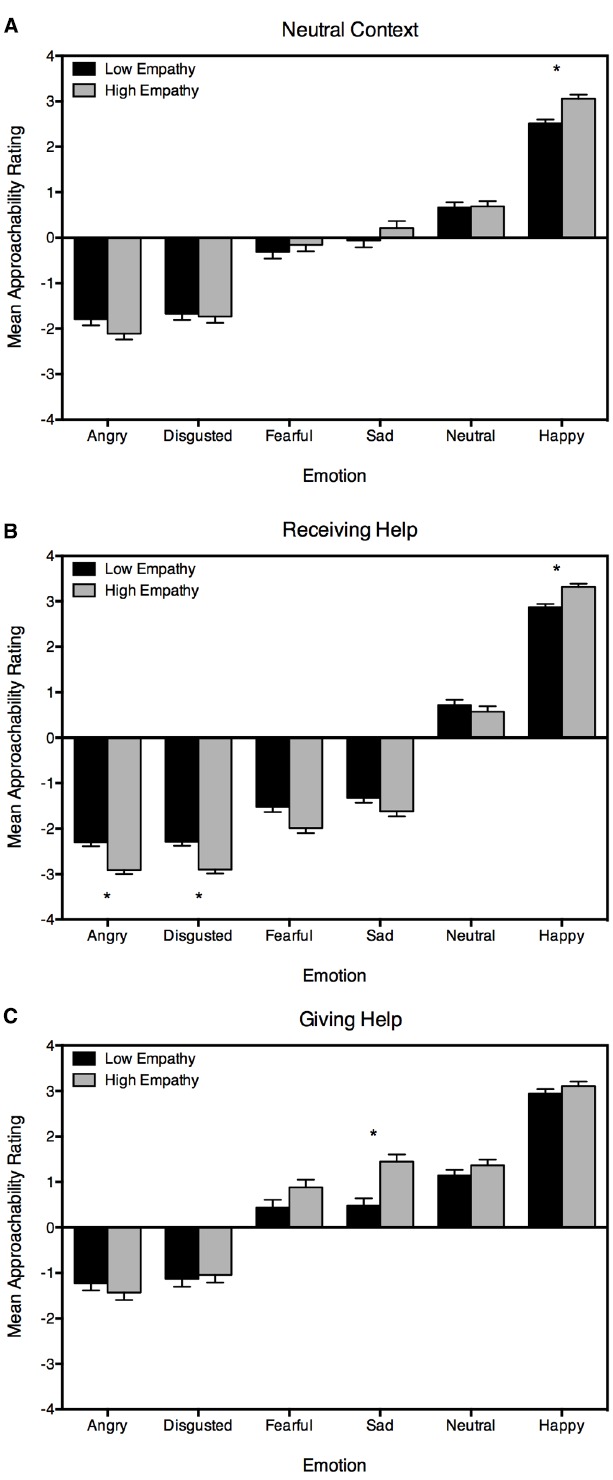
**Predicted approachability ratings for low and high empathy are displayed for each facial expression, separately for the three contexts: (A) neutral context, (B) receiving help, and (C) giving help.** Low empathy is estimated based on one SD below the mean and high empathy on one SD above the mean. Standard error bars are shown. Asterisks are provided to identify those emotions for which empathy was a significant predictor.

For the neutral context, the model was significant for angry, disgusted, sad, neutral and happy faces, but not fearful faces (see Table 2). As Table 2 indicates, empathy was a unique predictor of approachability judgments assigned to happy faces, with increased empathy associated with an increased willingness to approach happy faces (see Figure 1A). Intensity ratings predicted approachability judgments to disgusted and neutral faces. For disgusted faces, heightened perception of intensity predicted a reduced willingness to approach, whereas for neutral faces, heightened perception of emotional intensity predicted a greater willingness to approach. Threat ratings were a significant predictor of approachability judgments for angry, disgusted, sad, neutral and happy faces, such that heightened threat ratings predicted more negative approachability ratings for these expressions.

In the receiving help context, the model was significant for all emotions (see Table 3). Empathy was a unique predictor of approachability judgments assigned to angry, disgusted, and happy faces. Whereas heightened empathy predicted more negative approachability judgments to angry and disgusted faces, it predicted more positive approachability judgments to happy faces in this context (see Figure 1B). As Table 3 indicates, heightened intensity ratings predicted more negative approachability judgments for disgusted, fearful and sad faces, and more positive approachability judgments for happy faces. Heightened threat ratings were found to predict more negative approachability judgments for angry, disgusted, neutral, and happy faces.

**TABLE 3:** **Multiple regressions predicting approachability ratings in the receiving help context for each emotion**.

****	**Angry**				**Disgusted**				**Fearful**				**Sad**				**Neutral**				**Happy**			
**Model**	***F***	***df***	***p***	***R*^2^**	***F***	***df***	***p***	***R*^2^**	***F***	***df***	***p***	***R*^2^**	***F***	***df***	***p***	***R*^2^**	***F***	***df***	***p***	***R*^2^**	***F***	***df***	***p***	***R*^2^**
	21.51	3,116	<0.001	0.36	16.43	3,116	<0.001	0.30	5.31	3,116	0.002	0.12	2.83	3,116	0.041	0.07	9.59	3,116	<0.001	0.20	20.49	3,116	<0.001	0.35
**Predictor**	***B* (SE)**		**β**	***p***	***B* (SE)**		**β**	***p***	***B* (SE)**		**β**	***p***	***B* (SE)**		**β**	***p***	***B* (SE)**		**β**	***p***	***B* (SE)**		**β**	***p***
Intercept	–0.49 (0.36)				0.02 (0.41)				0.31 (0.55)				–0.11 (0.48)				1.08 (0.38)				2.40 (0.22)			
Empathy	–0.01 (0.00)		–0.21	0.007	–0.01 (0.00)		–0.17	0.035	0.00 (0.00)		–0.10	0.258	0.00 (0.00)		–0.06	0.517	0.00 (0.00)		0.01	0.929	0.01 (0.00)		0.24	0.003
Intensity	–0.10 (0.07)		–0.12	0.159	–0.10 (0.05)		–0.17	0.050	–0.32 (0.10)		–0.29	0.002	–0.23 (0.09)		–0.23	0.017	0.11 (0.06)		0.14	0.086	0.12 (0.04)		0.25	0.001
Threat	–0.25 (0.05)		–0.44	<0.001	–0.33 (0.08)		–0.37	<0.001	–0.06 (0.07)		–0.07	0.431	–0.05 (0.07)		–0.06	0.543	–0.38 (0.08)		–0.41	<0.001	–0.44 (0.08)		–0.42	<0.001

In the giving help context, the model was significant for angry, sad, neutral and happy faces (see Table 4). Empathy was a unique predictor of approachability judgments assigned to sad faces, with increased empathy associated with an increased willingness to approach sad faces to offer help (see Figure 1C). Intensity ratings predicted approachability judgments to angry, reflecting an increased willingness to approach angry faces to offer help when they were perceived to be more emotionally intense. Threat ratings were a significant predictor of willingness to approach and offer help to people displaying angry, neutral and happy faces, such that heightened perception of threat predicted less willingness to approach and offer help.

**TABLE 4:** **Multiple regressions predicting approachability ratings in the giving help context for each emotion**.

****	**Angry**				**Disgusted**				**Fearful**				**Sad**				**Neutral**				**Happy**			
**Model**	***F***	***df***	***p***	***R*^2^**	***F***	***df***	***p***	***R*^2^**	***F***	***df***	***p***	***R*^2^**	***F***	***df***	***p***	***R*^2^**	***F***	***df***	***p***	***R*^2^**	***F***	***df***	***p***	***R*^2^**
	7.47	3,116	<0.001	0.16	2.56	3,116	0.059	0.06	1.25	3,116	0.295	0.03	3.94	3,116	0.010	0.09	11.19	3,116	<0.001	0.22	7.49	3,116	<0.001	0.16

**Predictor**	***B* (SE)**		**β**	***p***	***B* (SE)**		**β**	***p***	***B* (SE)**		**β**	***p***	***B* (SE)**		**β**	***p***	***B* (SE)**		**β**	***p***	***B* (SE)**		**β**	***p***
Intercept	–0.65 (0.76)				0.54 (0.90)				0.71 (0.87)				–0.15 (0.70)				1.68 (0.40)						
Empathy	0.00 (0.01)		0.01	0.947	0.01 (0.01)		0.10	0.291	0.01 (0.01)		0.15	0.120	0.02 (0.01)		0.26	0.005	0.01 (0.00)		0.15	0.069	0.00 (0.00)		0.04	0.606
Intensity	0.32 (0.16)		0.20	0.044	–0.21 (0.11)		–0.19	0.055	–0.01 (0.16)		–0.01	0.927	0.13 (0.14)		0.09	0.349	0.07 (0.07)		0.09	0.272	0.10 (0.06)		0.14	0.101
Threat	–0.48 (0.10)		–0.46	<0.001	–0.19 (0.17)		–0.11	0.275	–0.17 (0.12)		–0.13	0.157	–0.14 (0.11)		–0.12	0.187	–0.45 (0.08)		–0.46	<0.001	–0.52 (0.13)		–0.35	<0.000

## Discussion

The current study examined the relationship between emotional empathy and context on judgments of approachability to emotional faces. Based on previous research it was hypothesized that greater empathy would be associated with more willingness to approach individuals in a giving help context when the facial expression signaled distress (i.e., sadness or fear). This hypothesis was partially supported. As expected, in the giving help context, heightened empathy predicted a greater willingness to approach sad faces to offer help. However, contrary to expectations, no such relationship was observed for fearful faces. We also anticipated that higher levels of empathy would be associated with more extreme approachability ratings to emotions across contexts. Our results indicated that increased sensitivity to emotion in individuals higher in empathy was not generalized, but specific to emotion and context. In the receiving help context, higher empathy predicted more negative ratings to certain negative emotions—specifically, angry, disgusted and fearful faces. Higher empathy also predicted a greater willingness to approach happy faces. Alternatively, in the giving help context, empathy only predicted approachability ratings to sad faces, with greater levels of empathy associated with a greater willingness to approach sad faces. In the neutral context, heightened levels of emotional empathy were associated with the perception of happy faces as more approachable. Collectively, these results indicate that the relationship between emotional empathy and approachability judgments to emotional faces is context specific. Heightened levels of emotional empathy predicted greater sensitivity to an individual’s facial expression when the perceiver was in need of help (i.e., in the receiving help context), which may reflect a response to a feeling of increased vulnerability in this state.

Importantly, the contribution of emotional empathy to approachability judgments across the three contexts was evident after controlling for threat and intensity ratings as predictors of approachability judgments. While in one specific circumstance (i.e., evaluation of approachability judgments to fearful faces in the receiving help context), threat and intensity ratings appeared to suppress the relationship between emotional empathy and approachability judgments. In all other instances, the relationship between empathy and approachability judgments did not change when threat and intensity ratings were included in the model as predictors. As threat and intensity were both associated with empathy in the current sample, this demonstrates that the relationship between empathy and approachability judgments is not solely driven by a heightened perception of threat and/or emotional intensity The findings demonstrate that emotional empathy is not a pervasive state that influences every social circumstance, but rather its influence is malleable, and context dependent. Our study illustrates the importance of considering situational context and empathy in combination—in demonstrating that empathy levels are associated with the approachability judgments we make, but this relationship is unique depending on the emotion and the context in which the emotion is encountered.

The results replicated previous findings of heightened sensitivity to emotional intensity in individuals with higher levels of empathy (e.g., Dimberg et al., 2011). Consistent with previous studies, higher levels of empathy were associated with higher ratings of emotional intensity, specifically to disgusted, fearful, sad, and happy faces. A novel finding that emerged in the current study was the evidence of heightened sensitivity to threat (i.e., angry, disgusted, and fearful faces) being associated with higher levels of empathy. This indicates that heightened emotional empathy appears to facilitate the detection of threat, and is not associated with a heightened perception of threat for all emotions, but rather a specific advantage for perceiving threat in those emotions that signal threat. It is notable that in the neutral context, a relationship between empathy and approachability ratings was only observed for happy faces. One explanation for this could be that perceived threat modulates this relationship between context and empathy levels. For instance, highly empathic individuals may be more sensitive to markers of direct threat (e.g., angry and disgusted faces)—in situations where they make themselves vulnerable (e.g., a receiving help context) but less sensitive to these cues in situations when the threat is mostly attributed to be relevant to another (e.g., giving help) or in a neutral context. It is possible that this increased sensitivity to threat accounts for the different relationship between approachability ratings and empathy that we observed in the receiving help context (e.g., how empathy was no longer a significant predictor of approachability judgments to fearful faces, once threat and intensity ratings were accounted for). This is a context where evaluation of threat is perhaps most pertinent, given that approaching others is necessary if assistance is to be received. In the other contexts, the decision to approach others may be seen more as a matter of choice, rather than a prerequisite need. In the current study, we did not assess the perception of threat in the different contexts; rather, we assessed the perceived threat of the faces independent of context. Future research investigating the particular relevance of threat specific to approachability judgments in distinct contexts may be able to provide greater insight into the effect of empathy observed in the current study.

An unexpected finding that emerged in the current study pertains to the fact that higher empathy predicted sad faces to be judged more approachable in the giving help context, but the same pattern was not observed for fearful faces. While both sadness and fear are considered “distress-related” emotions—emotions that signal helplessness with the presumed function of eliciting prosocial behavior—there are differences in the circumstances that typically elicit these two emotions. Sadness is thought to express a need for help that is less pressing than the emotion of fear, which signals immediate or impending danger in the environment (Marsh and Ambady, 2007). It is possible, then, in situations where the cause of the fear is unclear—such as in the giving help context where “dropping books” does not reflect a typical fearful circumstance—that the observer remains vigilant for threat cues, and the contribution of empathy is less apparent. Given that fear is thought to signal danger, it is logical that in a giving help scenario, where the observer has the choice to approach or avoid the expresser, that the observer will take less risks in selecting who to help. In addition, sadness appears to be a more congruent response to the scenario reported in the giving help context, and congruence of negative valence to a negative situation has been shown to be related to the degree of prosocial responding (Telle and Pfister, 2012). The proposed role of threat in the evaluation of fearful expressions in the giving help context is supported by the fact that heightened sensitivity to threat in fearful faces was associated with higher empathy levels. Unexpectedly, recognition of fear was not associated with empathy. This may indicate that the task assessing facial expression recognition was not sensitive enough to detect subtle differences in facial recognition abilities, particularly since it has been on more demanding facial expression recognition tasks that differences in fear recognition have been demonstrated (Besel and Yuille, 2010).

It is important to note some limitations of the current study. Our sample consisted of only females, which limits the generalizability of the results. Sex differences in emotional empathy, categorized by higher levels of empathy in women, have been systematically documented in the literature (for a review, see Eisenberg and Lennon, 1983). Whether these result from biological underpinnings or are largely due to social conditioning remains a matter of contention (Hermans et al., 2006). Further application of our paradigm to male samples is necessary, to examine whether the results are generalizable to the population at large or if different patterns emerge by sex. In addition, the measure used to assess empathy in this study was a self-report scale. While the QMEE scale has been used extensively in the literature as a measure of emotional empathy (Dimberg et al., 2011; Tibi-Elhanany and Shamay-Tsoory, 2011), it would be of interest to examine whether the different relationships between empathy and approachability judgments between contexts translate to differences in physiological responding. Using a Swedish translation of the QMEE, individuals higher in empathy were found to spontaneously and rapidly mimic happy and angry facial expressions, whereas the low empathy group did not display physiological differences between happy and angry faces (Dimberg et al., 2011). In view of our results, an interesting avenue for future research may be to examine the extent to which physiological responding (specifically, facial mimicry) may underpin individual differences in approachability judgments related to empathy. That is, are these judgments driven by physiological emotional responses to emotion in others that then subsequently drive social decision-making?

One further consideration is that individual differences in emotional empathy may be modulated by other psychological variables (e.g., depression and anxiety; Negd et al., 2011; Schreiter et al., 2013). Therefore, it is possible that our documented findings were not just relevant to individual differences in emotional empathy in isolation but may be influenced by individual characteristics that we did not assess in this initial study. While establishing that empathy is an individual characteristic that predicts approachability judgments is a step forward in recognizing the role of individual differences in making social judgments, future investigations should investigate the significance of other individual differences, and their relationship to empathy, if a comprehensive understanding of real-life decision-making on approachability is to be achieved. We also note that the tasks used in the current study involve very specific contextual scenarios. These tasks were chosen due to their previous use in approachability research and perceived ecological validity, however it is possible that subtle differences in the scenarios can influence the documented results. Replication of this study with a broader range of scenarios, such as tasks that vary in content and congruency to context, would allow a more detailed understanding of how empathy influences approachability judgments made under certain conditions.

The central finding of our research was that the relationship between empathy and approachability judgments assigned to emotional faces was specific to emotion and context. Higher emotional empathy is not only associated with an increased likelihood that an individual will help someone displaying the facial expression of sadness, but it is also associated with the perception of approachability when receiving help, such that individuals higher in empathy have more extreme approachability judgments to angry, disgusted, and happy faces. What is novel about this finding is that it indicates that emotional empathy does not have a pervasive influence on sensitivity to facial expressions, but is pertinent to certain situations, such as when the individual is vulnerable (i.e., when receiving help), or when the need for help is observed. This is particularly relevant if we are to apply this understanding of the behavioral outcomes of lower levels of empathy (e.g., less sensitivity to threat when seeking help) to populations in which empathy-related deficits have been observed, such as those with psychopathy and autism spectrum disorder (see, e.g., Clark et al., 2008; Decety et al., 2013).

Our work further expands our understanding of the relationship between emotional empathy and facial expression processing, in demonstrating that emotional empathy is associated with enhanced detection of threat, with a specific advantage for perceiving threat in those emotions that convey direct and indirect threat (i.e., anger, disgust, and fear). In addition, this work extends on previous approachability research (Porter et al., 2007; Willis et al., 2011b), by demonstrating not only the critical role of facial expression interpretation, but the contribution of individual and contextual factors in such evaluations. Our findings are the first to demonstrate that levels of empathy predict approachability judgments over and above threat and intensity ratings. Further examination of the manner through which empathy may modulate these social decision-making processes may provide insight into how these processes are disrupted in special populations, and how these deficits can be addressed to assist with interpretation of social cues, a process critical to accurate and appropriate social decision making in everyday life.

### Conflict of Interest Statement

The authors declare that the research was conducted in the absence of any commercial or financial relationships that could be construed as a potential conflict of interest.

## Footnotes

1 Simple linear regressions were also run to examine whether empathy was a significant predictor of approachability ratings (by emotion and context), independent of threat and intensity. There were no changes to the significance of empathy a sole predictor, compared to when it was included as a predictor in the multiple regression analyses, with one exception. Empathy was a significant sole predictor of approachability ratings assigned to fearful faces in the receiving help context, *F*(1,118) = 4.41, *p* = 0.038, *R*^2^ = 0.036, *B* (SE) = –0.01 (0.00), β = –0.19, but was no longer a significant predictor when the additional variables were included (see Table 3).
